# Interference of Large Clostridial Glucosyltransferases with the Endolysosomal Pathway: Toxin-Induced Imbalance of Early Endosomes, Functional Lysosomes and Autophagosomes

**DOI:** 10.3390/toxins18040186

**Published:** 2026-04-15

**Authors:** Anna Langejürgen, Gudula Schmidt, Leon Unsöld, Helma Tatge, Ethel Oyson, Ralf Gerhard

**Affiliations:** 1 Institute of Toxicology, Hannover Medical School, 30625 Hannover, Germany; langejuergen.anna@mh-hannover.de (A.L.); tatge.helma@mh-hannover.de (H.T.); ethel.oyson@stud.uni-hannover.de (E.O.); 2 Institute for Experimental and Clinical Pharmacology and Toxicology, University of Freiburg, 79104 Freiburg, Germany; gudula.schmidt@pharmakol.uni-freiburg.de

**Keywords:** Clostridioides difficile toxins, endosome, lysosome, pore formation, Rab7, transcription factor EB

## Abstract

Toxin A and B from Clostridioides difficile are the main pathogenicity factors for clinical symptoms of C. difficile infections. Receptor-mediated endocytosis and endosomal escape are required for targeting substrate proteins of the Rho-GTPase family. We previously reported that Toxin B (TcdB) affects endo-lysosomal transport and autophagic flux of target cells. These effects are independent from pathogenic Rho inhibition. Here, we aimed at further characterization of this event by immunofluorescent characterization of the vesicular structures that are affected. We found large aggregates of damaged endolysosomal structures positive for EEA1, LAMP1, CHMP4B and TcdB, as well as an increase in perinuclear concentration of non-mature autophagosomes (amphisomes) positive for SQSTM, Rab7, and LC3B. We investigated whether Rab7, a regulator of late endosome transport, is causative for decreased lysosome function. Although TcdB induced an increase in active Rab7, as tested by an RILP pull-down assay, inhibition of Rab7 did not prevent TcdB-induced decrease in cathepsin D as a surrogate for lysosome dysfunction. It also indicates that the observed increase in Rab7 positive amphisomes is secondary to lysosomal dysfunction. By applying an autoproteolytic deficient mutant of TcdB we proved that the release of the glucosyltransferase domain is mandatory for triggering all of these effects. This suggests that after membrane perforation the toxin remnants leave an open leak in endolysosomes affecting ion homeostasis. Investigation of all large clostridial glucosyltransferases and other toxins revealed lysosomal dysfunction as a general effect of many but not of all toxins that integrate into the endosome membrane.

## 1. Introduction

Pathogenic Clostridioides difficile are Gram-positive bacteria that produce protein toxins A (TcdA) and B (TcdB) [1]. TcdA and TcdB represent the primary virulence factors in C. difficile infections, with symptoms ranging from mild diarrhea to pseudomembranous colitis [1,2]. TcdB is a member of the large clostridial glucosylating toxins (LCGT) family. Classified as an AB toxin, TcdB comprises distinct domains for its uptake into target cells and pathogenic effect. A glucosyltransferase domain (GTD) mediates the main pathogenic effect. The segment responsible for binding, endocytosis and transport is composed of a cysteine protease domain (CPD), a receptor binding/delivery domain (RBDD), and the combined repetitive oligopeptides (CROPs) [Gerhard] [3].

Following receptor binding, TcdB is internalized by receptor-mediated endocytosis [4,5]. Subsequently, endosomal acidification triggers a conformational change in TcdB, exposing a hydrophobic transmembrane domain (TMD), which interacts with the endosomal membrane to facilitate membrane integration [6]. Membrane integration enables the translocation of the two enzymatically active toxin domains: the CPD and the GTD. On the cytosolic membrane surface, inositol hexakisphosphate (InsP6) activates the CPD, which then autoproteolytically releases the GTD at a cleavage site behind L543 [7]. The trunk of TcdB is assumed to remain integrated into the endosomal membrane and thus, dedicated to lysosomal degradation. Within the cytosol, the released GTD catalyzes mono-glucosylation of Rho GTPases, which are molecular switches that regulate the actin cytoskeleton. Glucosylation inactivates Rho GTPases, leading to disorganization of the actin cytoskeleton, which results in cell rounding and eventual apoptosis.

Besides the canonical Rho-inactivation by all large clostridial glucosyltransferases, high concentrations of TcdB additionally induce early cell death by triggering reactive oxygen species (ROS) generation. Inhibition of NADPH oxidase or ROS scavenging prevents this early cell death [8]. This specific effect is caused by Ca^2+^-flux from endosomes and can be abolished by inhibitors of L-type Ca^2+^-channels such as nifedipine [8]. The precise mechanism that results in the aforementioned process has not been investigated so far. We hypothesize that integration into the endosomal membrane affects membrane potential, thereby activating voltage-dependent ion channels. We previously demonstrated that TcdB impairs lysosomal function and, consequently, affects the autophagic flux, presumably by the same mechanism [9,10]. Notably, inhibition of L-type Ca^2+^-channels did not rescue lysosomal dysfunction, indicating distinct downstream processes, likely based on the same mechanism, i.e., interference with membrane integrity. TcdB impairs lysosomal function independently from Rho glucosylation, as shown by the application of a glucosyltransferase-deficient mutant TcdB. Apparently, toxin embedding into the endosomal membrane interferes with physiological endolysosomal processes. Vesicular injury has been established to mediate their autophagic clearance or repair, depending on the severity [11]. Severe membrane rupture reveals the luminal side of the vesicular membrane, displaying ß-galactosides and triggering a galectin response resulting in macroautophagy of the affected structure [12,13,14]. We previously excluded severe membrane rupture of the endosomal membrane since TcdB does not elicit a typical galectin-3/-8 response in HEp-2 cells [9]. Conversely, the ESCRT-III complex containing charged multivesicular body protein 4 (CHMP4) is responsible for repair of minor membrane damage [11]. Notably, the assembly of the ESCRT machinery does not require gross membrane rupture, as Ca^2+^ efflux suffices to recruit it [15].

By analyzing marker proteins for late endosomes (Rab7), lysosomes (LAMP1, cathepsin D), amphisomes/autophagosomes (LC3B, SQSTM1) and endosomal damage (CHMP4B) we aimed at characterizing imbalance in endolysosomal vesicles and affected autophagosomes induced by TcdB. We further analyzed activation of Rab7 to detect possible interference with late endosomal transport by TcdB and used different mutants of TcdB to specify the structural requirements for this phenomenon.

## 2. Results

### 2.1. TcdB Reduces Cathepsin D in a Time- and Concentration-Dependent Manner

To focus on toxin integration into endosomal membranes we used glucosyltransferase-deficient TcdB NXN, avoiding bias by Rho inactivation. Treatment with TcdB NXN does not induce cell rounding, allowing us immunofluorescence staining of relevant targets with cells sustaining unchanged morphology.

**Figure 1:**
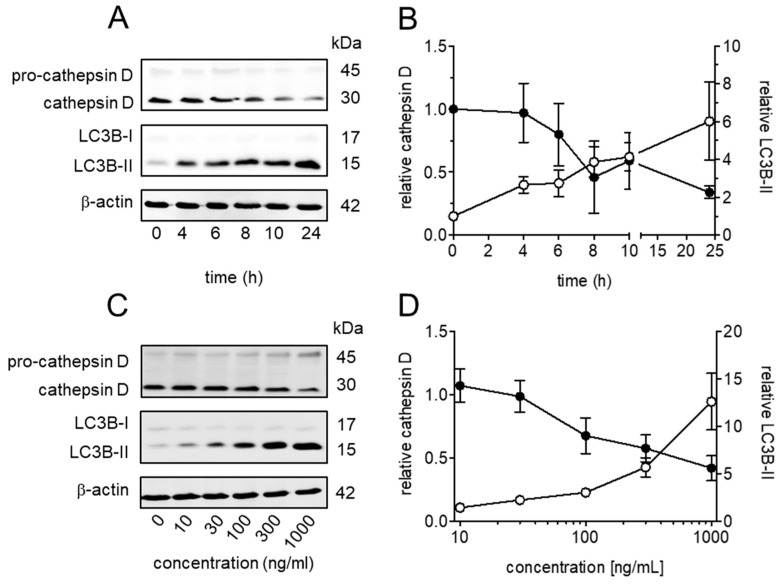
Cathepsin D and LC3B-II are affected by TcdB in a time- and concentration-dependent manner. (**A**) Representative immunoblots show a time-dependent decrease in mature cathepsin D (30 kDa) and increase in LC3B-II (15 kDa) after treatment of HEp-2 cells with 1 µg/mL TcdB NXN for 24 h. (**B**) Densitometrical evaluation of cathepsin D and LC3B-II relative to β-actin from six separate experiments. (**C**) Representative immunoblots show mature cathepsin D (30 kDa) and LC3B-II (15 kDa) after treatment of HEp-2 cells with the indicated concentrations of TcdB NXN for 24 h. (**D**) Densitometrical evaluation of cathepsin D and LC3B-II ratio with β-actin compared to untreated controls from six separate experiments. Shown are mean values ± SE, n = 6, resulting from three independent experiments in duplicate.

We previously observed the effect of TcdB or TcdB NXN on the abundance of the lysosomal marker protein LAMP-1 [9,16]. However, the LAMP-1 signal exhibited great variability and did not allow for discrimination between function and number of lysosomes. We therefore aimed at validating a more reliable marker for alterations in lysosomal function. Western blot analyses revealed a time-dependent decrease in the mature lysosomal protease cathepsin D in HEp-2 cells after treatment with TcdB NXN (Figure 1A). Processing pro-cathepsin D to mature cathepsin D requires acidic pH and lysosomal protease activity [17]. TcdB NXN-mediated reduction in cathepsin D thus correlates with a decrease in lysosomal function. In accordance with decreased lysosomal function, immunoblots showed an increase in autophagosome marker LC3B-II hinting at impaired autophagic flux in response to TcdB NXN treatment. Time course showed an inverse correlation with continuous decrease in cathepsin D and increase in LC3B-II levels over the observed period of 24 h. TcdB-induced changes in cathepsin D and LC3B-II levels were also affected in a concentration-dependent manner as measured after 24 h of exposure (Figure 1C). The cathepsin D signal reduced to approximately 50% at 300 ng/mL (~1 nM) TcdB NXN, while LC3B-II signals increased 10 to 15-fold at the highest concentration of TcdB NXN (1 µg/mL). To validate immunoblot findings, cathepsin D activity was additionally measured using an MCA-based assay. A decrease in cathepsin D activity by 40% confirmed the decrease in functional protease levels (Figure S1). Hence, cathepsin D served as a valid marker to evaluate TcdB-induced lysosomal dysfunction.

### 2.2. Alteration of Endosomal and Autophagic Vesicles in TcdB NXN-Treated Cells

**Figure 2:**
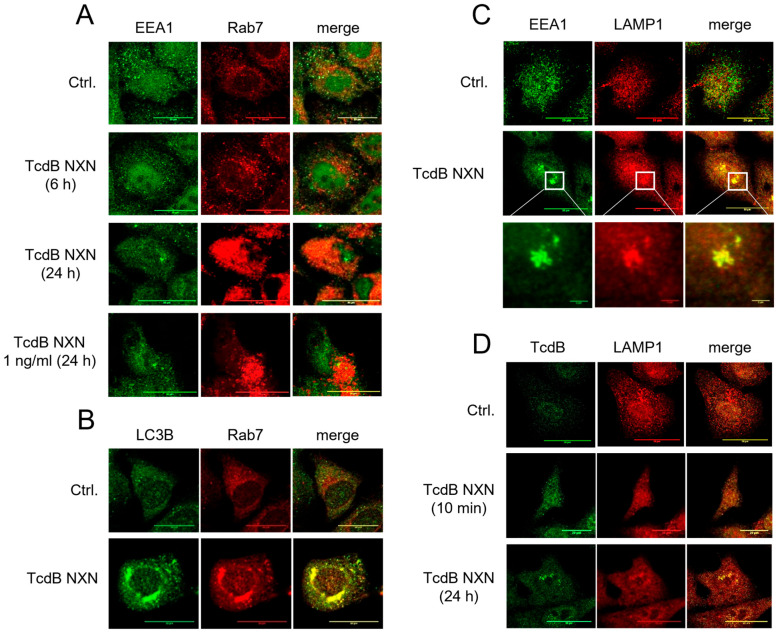

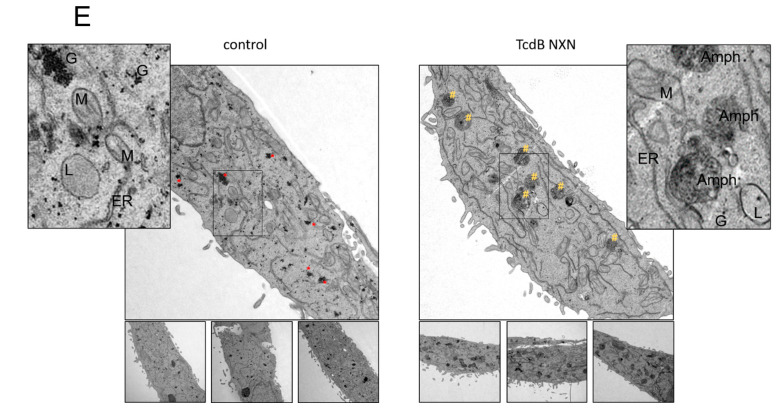
TcdB affects marker proteins for early and late endosomes as well as lysosomes. (**A**) HEp-2 cells treated with 1 µg/mL TcdB NXN for the indicated times were immunofluorescently stained for early endosome marker EEA1 (green) and late endosome marker Rab7 (red). Treated cells showed a clear decrease in peripheral EEA1 staining and increase in Rab7 positive structures compared to untreated controls. EEA1 and Rab7 did not colocalize as shown in merged images. The change in EEA1and Rab7 level was also visible at a concentration of 1 ng/mL toxin. (**B**) Immunofluorescence microscopy reveals that TcdB NXN-induced increase in Rab7 (red) colocalized with LC3B (green), indicative for immature autophagosomes/amphisomes. (**C**) In addition to an increase in amphisomes, TcdB NXN also induced aggregated vesicular structures in close proximity to the nucleus after 24 h. These structures were positive for EEA1 and LAMP1 as markers for late lysosomes. (**D**) Staining for TcdB revealed that the EEA1/LAMP1 positive aggregates were also positive for TcdB NXN. (**A**–**D**) show representative images for at least three separate experiments in duplicate. (**E**) Transmission electron microscopy of HEp-2 cells treated with 1 µg/mL TcdB NXN for 24 h. Untreated controls (left compilation) show normal structures of mitochondria (M), lysosomes (L) and endoplasmic reticulum (ER) (see magnification of indicated region). Furthermore, electron-dense spots indicate glucagon depots (G in magnification; red asterics in overview) within the cytosol. In TcdB NXN-treated cells, glucagon depots decreased and electron-dense vesicles appeared (yellow hashtag). These vesicles show indifferently structured content and lack a double membrane (see magnification) and were considered as amphisomes (Amph). Images of three further examples from respective groups prove that amphisomes are a general and characteristic effect of TcdB.

To investigate vesicular trafficking upstream of lysosomes, we next visualized early and late endosomes by immunofluorescence staining of early endosome antigen 1 (EEA1) and Rab7, respectively. Immunofluorescence imaging revealed a profound effect of TcdB NXN on the endosomal system. Untreated cells exhibited an even distribution of EEA1 throughout the periphery (Figure 2A). TcdB NXN-treated cells showed a time-dependent reduction in peripheral EEA1 and perinuclear EEA1 aggregation after 24 h treatment (Figure 2A). Following 6 h of treatment with 1 µg/mL TcdB NXN, EEA1 puncta grouped near the nucleus, whereas the peripheral signal decreased. After 24 h of exposure, TcdB NXN induced aggregation of perinuclear EEA1, while peripheral EEA1 signal was strongly reduced.

In contrast to EEA1, Rab7 signal increased progressively after 6 to 24 h of exposure to 1 µg/mL TcdB NXN (Figure 2A). EEA1 aggregates and Rab7 signal did not co-localize. Notably, HEp-2 cells exhibited EEA1 aggregation and increase in Rab7 signal even when treated with only 1 ng/mL toxin, highlighting the sensitivity of the marker proteins. The TcdB NXN-mediated phenotype therefore comprises two distinct endosomal populations, which progressively aggregate and accumulate, respectively.

Therefore, TcdB NXN treatment induced remarkably diverging responses from early and late endosomal markers. Beyond late endosomes, Rab7 localizes to lysosomal and autophagosomal vesicles as well [18]. Since immunoblots showed an increase in LC3B-II, Rab7 signal may correlate with the accumulating autophagosomal marker. Indeed, co-staining for Rab7 and LC3B showed co-localization of these two marker proteins (Figure 2B). Aligning with the pattern observed for Rab7, perinuclear LC3B signal increases following TcdB NXN exposure.

TcdB NXN-induced EEA1-positive aggregates were also positive for LAMP1 (Figure 2C). A zoom-in showed the aggregates being composed of single, large EEA1 and LAMP1-positive puncta, resembling vesicular structures. Since LAMP1 is not exclusively located at lysosomes, we additionally stained cells for cathepsin D and found aggregates near the nucleus also positive for cathepsin D but not for Rab7 (Figure S2). Interestingly, TcdB NXN localized to the EEA1/LAMP1-positive aggregates following 24 h of exposure (Figure 2D). Using a house-made TcdB antibody, we were able to track TcdB NXN endocytosis, which eventually resulted in the TcdB NXN-positive aggregates. Notably, the non-cleavable TcdB NXN mutant was not detectable in the cell following 24 h of treatment (Figure S3).

To visualize the TcdB NXN-mediated phenotype in more detail, we performed TEM on TcdB NXN-treated HEp-2 cells. TcdB NXN induced an increase in electron-dense large vesicles (Figure 2E). Their number, size and localization suggest that these vesicles represent the SQSTM/LC3B/Rab7-positive autophagosomes.

### 2.3. Autoproteolysis of TcdB Is Essential for Lysosomal Impairment and Endosomal Alterations in HEp-2 Cells

**Figure 3:**
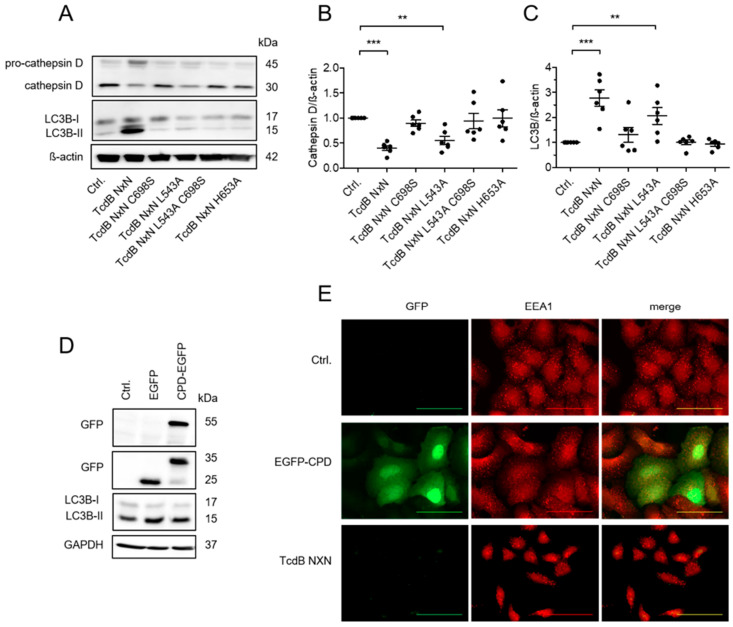
Autoproteolytic release of the GTD is required for persistent disturbance of endolysosome function. (**A**) Representative immunoblots showing mature cathepsin D (30 kDa) and LC3B-II (15 kDa) after treatment of HEp-2 cells with 1 µg/mL of TcdB NXN or its various non-cleavable mutant forms C698S, L543A, H653A, or the double mutant L543A/H653A. (**B**,**C**) Densitometrical evaluation of six separate experiments show that only cleavable TcdB NXN and poorly cleavable TcdB NXN L543A significantly decreased cathepsin D level and increased LC3B-II level. TcdB NXN for 24 h. Shown are mean values ± SE; n = 6 resulting from three separate experiments in duplicate. (**D**) Representative immunoblots showing LC3B level after transfection of HEp-2 cells with the cysteine protease domain of TcdB (amino acids 543−800). Expression of EGFP or EGFP-CPD in transfected cells was verified by immunoblot against EGFP for EGFP alone or for EGFP-CPD (35 kDa). (**E**) Immunofluorescence staining of EGFP or EEA1 revealed lack of effect of EGFP-CPD on endosome marker EEA1 compared to non-transfected controls. Treatment with TcdB NXN served as positive control for effect on EEA1. Figures are representative for two independent experiments. ** and *** indicate significances of *p*-values < 0.01. and <0.001, respectively.

As previously reported, non-cleavable toxin mutants TcdB NXN C698S or TcdB NXN L543A show different effects on lysosomal marker LAMP1 or LC3B-II [9]. Ablation of the cysteine protease activity by exchange of the essential cysteine at position 698 completely abolished the TcdB NXN-induced decrease in cathepsin D; while mutation of the cleavage site (L543A) only partially reduced this effect (Figure 3A, Figure S1). It remains to be determined whether the observed effect (i) depends on protease activity or if (ii) the mutated cleavage site alters the topology of the toxin within the endosomal membrane, or if (iii) only the cleaved toxin interferes with transporters thereby indirectly affecting ion homeostasis without pore formation. To dissect this, we additionally generated a double mutant of TcdB (TcdB NXN L543A, C698S) as well as TcdB NXN H653A. Histidine-653 is part of the essential triad of amino acids for protease activity; its exchange thus inhibits proteolytic activity [19]. Immunoblots revealed that only complete inhibition of the catalytic activity as in TcdB NXN C698S, or TcdB NXN H653A, abolished the effect on markers cathepsin D and LC3B-II (Figure 3B,C). Mutation of the cleavage site (TcdB L543A) was not sufficient to prevent TcdB-induced lysosomal impairment completely, probably due to residual autoproteolytic cleavage.

To rule out any effect of the active CPD itself, we transfected cells with eGFP-tagged CPD. Transfection with eGFP-CPD did not alter levels of LC3B-II as the most sensitive marker for the TcdB NXN-induced effect in HEp-2 cells (Figure 3D). EGFP-CPD transfection was also evaluated using immunofluorescence imaging (Figure 3E). The eGFP-positive HEp-2 cells containing the eGFP-tagged CPD exhibited EEA1 distribution comparable to the untreated control, while TcdB NXN-treated cells revealed the characteristic reduction in peripheral EEA1, with EEA1 aggregating near their nuclei instead. These findings suggest that the cysteine protease activity itself is not causative for TcdB NXN-induced lysosomal dysfunction.

### 2.4. TcdB Perforates Endosomal Membrane to Deliver Its GTD to the Cytosol: Correlation with Impairment of Lysosomes

**Figure 4:**
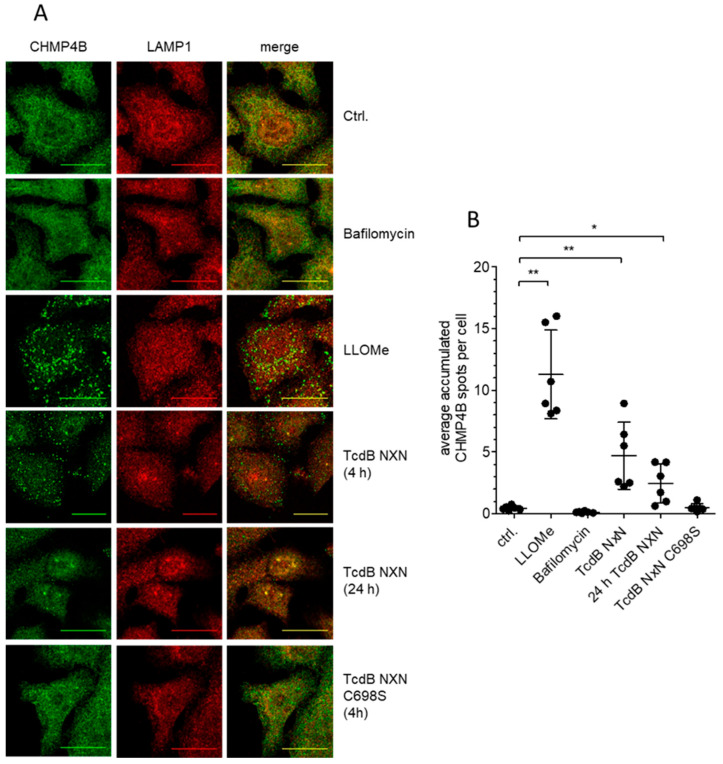
CHMP4B accumulates in response to TcdB remnants in endosomes. (**A**) Immunofluorescent staining against LAMP1 (red) and CHMP4B (green) as a marker for moderate endosome membrane damage showed accumulation of CHMP4B on endosomes of HEp-2 cells after treatment with positive control LLOMe (1 mM) as well as with 1 µg/mL TcdB NXN. Neither non-cleavable TcdB NXN C698S nor bafilomycin provoked CHMP4B accumulation. CHMP4B response was observed after 4 h treatment with TcdB NXN and barely visible after 24 h treatment. (**B**) Quantification of CHMP4B spots per cell from six separate experiments. Refering to background signal in untreated controls, CHMP4B positive granules of LLOMe sample were used to set the threshold for pixel intensity to identify specific structures. All images from one experiment were analysed with the same setting. * and ** indicate significances of *p*-values < 0.05. and <0.01, respectively.

Previous results excluded severe damage of the endosomal membrane after TcdB NXN exposure [9,20,21]. If, however, TcdB NXN causes minor damage to the endosomal membrane without exposing luminal ß-galactosides to the cytosolic leaflet, it would instead promote activation of the cell’s membrane repair system. CHMP4B is part of the ESCRT-III system in charge of membrane repair, which is recruited upon Ca^2+^ efflux out of endolysosomal vesicles [15]. We utilized the lysomotropic agent L-leucyl-L-leucine methyl ester (LLOMe) to visualize endolysosomal membrane damage as a positive control for CHMP4B accumulation, indicative of the initiation of membrane repair. LLOMe is transported to acidic vesicles where it undergoes cathepsin C-dependent condensation leading to membrane rupture [22]: Indeed, cells treated with LLOMe or TcdB NXN exhibited granulation of CHMP4B signal, indicative for sites of membrane damage (Figure 4A). TcdB NXN-induced CHMP4B accumulation was less pronounced compared to LLOMe. Accumulation of CHMP4B, indicated by the enlarged puncta, was more evident after 4 h than after 24 h of TcdB NXN treatment, indicating either partial repair or partial degradation of damaged endosomes. In comparison, untreated controls as well as bafilomycin A1-treated cells showed an even signal distribution without noticeable accumulation of CHMP4B (Figure 4A). TcdB NXN-induced CHMP4B-positive structures were also LAMP1-positive, indicating that CHMP4B accumulated on vesicles of the endolysosomal pathway. In line with cathepsin D and LC3B-II results, non-cleavable TcdB NXN H653A did not alter CHMP4B signal distribution (Figure 4A,B), pointing out that non-cleavable TcdB NXN variants do not activate ESCRT-III recruitment. Absence of ESCRT-III response suggests that the GTD, if not released by autoproteolysis, seals the TcdB-formed endosomal leak, thereby putatively preventing ion efflux. As a positive control for dysregulation of endosomal homeostasis, bafilomycin A1 was applied to prevent endosomal acidification by inhibiting vATPase [23]. Notably, bafilomycin did not alter CHMP4B distribution, while producing a similar phenotype regarding endosomal, lysosomal and autophagosomal markers like TcdB NXN. These findings highlight the mechanistic difference between functional impairment and structural damage to endolysosomal vesicles. Notably, in contrast to early cell death triggered by TcdB-induced ROS production [8,24,25], CHMP4B accumulation was not nifedipine sensitive (Figure S4).

### 2.5. Disturbance of Endosomal Ion Homeostasis Affects TFEB Activity

Lysosomal dysfunction results in clearance of terminally damaged structures and enhanced biogenesis of replacement lysosomes. The transcription factor EB (TFEB) regulates the transcription of lysosomal protein genes necessary for lysosomal biogenesis [26]. The mechanistic target of rapamycin complex 1 (mTORC1) phosphorylates TFEB, mediating its retention within the cytosol. Impairment of lysosomal structure or function promotes dephosphorylation of TFEB, associated with nuclear translocation and transcriptional activity.

**Figure 5:**
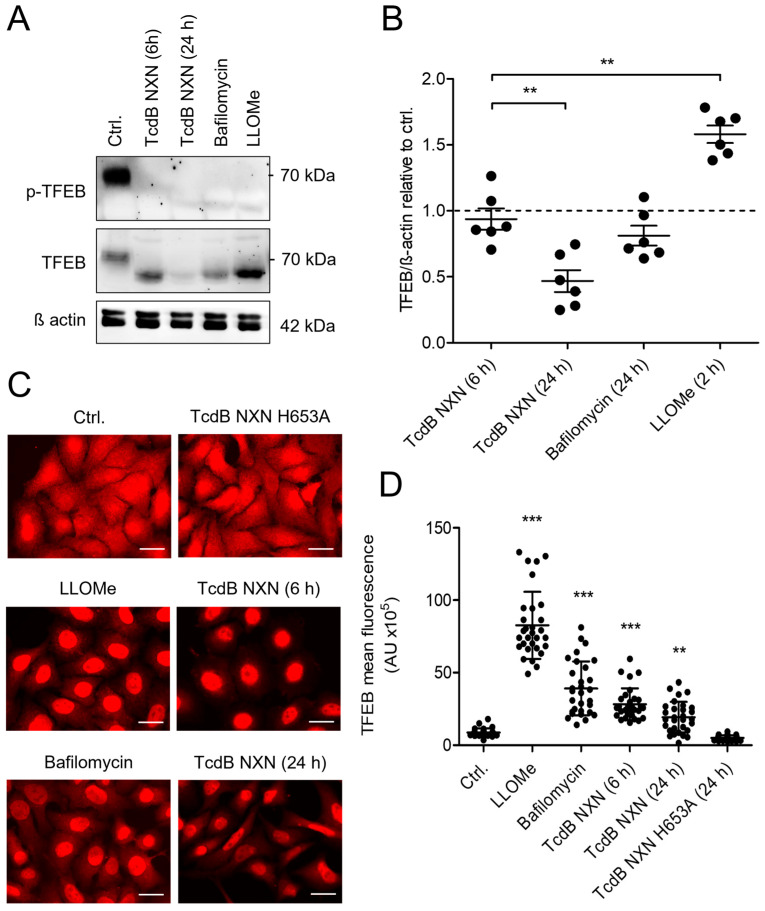
Transcription factor EB is activated in response to TcdB. (**A**) Level of phosphorylated TFEB (p-TFEB, 70 kDa) and total TFEB are shown by representative immunoblot analyses of HEp-2 cell lysates. TcdB NXN as well as bafilomycin A1 or LLOMe-induced decrease in level of p-TFEB (70 kDa). Accordingly, the apparent molecular weight of TFEB appeared to be <70 kDa compared to untreated controls. β-Actin served as the loading control. (**B**) Densitometrical evaluation of blots showing total TFEB from six separate experiments reveal that TcdB NXN significantly reduces level of TFEB, whereas LLOMe significantly increases TFEB level (mean values ± SE, n = 6). (**C**) Immunofluorescence of TFEB localization in HEp-2-cells shows decreased cytosolic TFEB and a predominantly nuclear localization. (**D**) Quantification of nuclear TFEB. For quantification, a defined ROI (5 µm × 5 µm) was used to measure mean pixel intensity within the area of all nuclei. Ten randomly chosen nuclei from three separate experiments were analyzed (mean values ± SE, n = 30). ** and *** indicate significances of *p*-values < 0.01. and <0.001, respectively.

Immunoblots revealed that treatment of cells with either TcdB NXN, bafilomycin or LLOMe induced a reduction in levels of phosphorylated TFEB, compared to the untreated control (Figure 5A). Total TFEB signal exhibited different migration characteristics in SDS-PAGE correlating with its phosphorylation status [27]. While LLOMe induced an increase in TFEB levels, cells treated with TcdB NXN for 6 h exhibited a TFEB signal comparable to the untreated control. Cells exposed to bafilomycin for 24 h also exhibited TFEB levels comparable to the untreated control. However, 24 h of TcdB NXN exposure significantly reduced total TFEB levels (Figure 5B). Prolonged treatment with TcdB NXN either downregulated TFEB expression, led to rapid TFEB degradation or impaired its detection by the antibody. Furthermore, immunostainings revealed nuclear translocation of TFEB upon treatment with TcdB NXN, Bafilomycin or LLOMe, consistent with the decreased phosphorylation status (Figure 5C). Non-cleavable TcdB NXN H653 had no effect on TFEB translocation, further supporting its role as an appropriate negative control for elucidating the mechanism of toxin-induced endolysosomal dysfunction. TcdB-induced translocation of TFEB into the nucleus was not affected by nifedipine, indicating that L-type voltage-dependent calcium channels are not significantly involved (Figure S5).

After exposure to LLOMe or TcdB NXN for 6 h, cells exhibited increased nuclear TFEB signal (Figure 5D). On average, treatment with TcdB NXN and bafilomycin for 24 h mediated a modest increase in nuclear TFEB signal, compared to the untreated control. Consistently, treatment with bafilomycin or TcdB NXN led to a decrease in peripheral TFEB signal relative to the untreated control. However, the peripheral TFEB signal after prolonged TcdB NXN or bafilomycin exposure was still higher than in LLOMe-treated cells, suggesting a time-dependent regulation of TFEB activity [28].

### 2.6. Lysosomal Dysfunction Is Associated with Rab7 Upregulation

**Figure 6:**
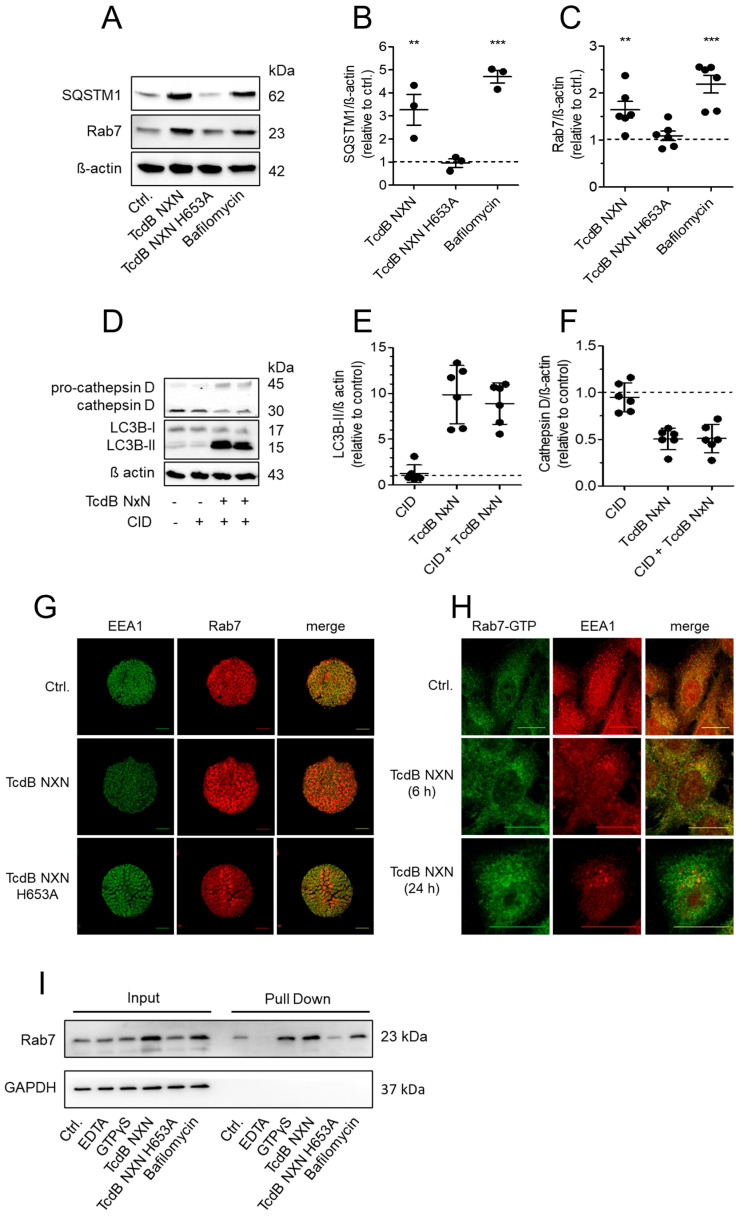
TcdB induces upregulation of autophagic marker SQSTM1 and Rab7. (**A**) Immunoblot against autophagic marker SQSTM1 and Rab7 in Hep-2 cells treated with TcdB NXN, non-cleavable TcdB NXN H653A, or bafilomycin A1. Β-Actin served as the loading control. (**B**,**C**) Densitometrical evaluation of SQSTM and Rab7 blots from three or six separate experiments, respectively. (**D**) Representative immunoblots of cathepsin D and LC3B of Hep-2 cells treated with TcdB NXN and TcdB NXN in combination with the Rab7 inhibitor CID106220 (40 µM). (**E**,**F**) Densitometrical evaluation of six immunoblots as shown in D (mean values ± SE, n = 6). Inhibition of Rab7 did not reduce the TcdB NXN effect on cathepsin D and LC3B. (**G**) The toxin effect on EEA1 and Rab7 as a marker for early endosomes and late endosomes/autophagosomes/amphisomes was also tested in murine intestinal organoids. TcdB NXN but not non-cleavable TcdB NXN reduced peripheral EEA1 and increased Rab7 level as shown by immunofluorescent staining. (**H**) Immunofluorescence staining of Rab7-GTP (green) specifically revealed an increase in active Rac1 in TcdB NXN-treated Hep-2 cells along with decreased EEA1 (red). (**I**) Effect of TcdB NXN on the activity status of Rab7 (Rab7-GTP) was additionally tested in pull-down assays. An immunoblot representative for three separate experiments showed the level of Rab7 in input and in precipitates after binding to immobilized effector protein RILP. Cells treated with TcdB NXN as well as with bafilomycin A1 showed higher levels of Rab7 in cell lysates and, accordingly, higher levels in pull-down experiments. EDTA and GTPγS served for inactivation and activation of Rab7, respectively, for negative and positive controls in pull-down assays. ** and *** indicate significances of *p*-values < 0.01. and <0.001, respectively.

Our findings point to interference of TcdB NXN with late endosomes and endolysosomes, which in turn affects processing of autophagosomes. In line with LC3B-II results, immunoblots showed an increase in SQSTM1, which is indicative for accumulation of autophagosomes. Likewise, Rab7, which is also present on autophagosomes, increased after TcdB NXN treatment (Figure 6A). The same effect was induced by bafilomycin, which increased SQSTM1 and Rab7 levels comparably to TcdB NXN, while non-cleavable TcdB NXN H653A did not significantly alter SQSTM1 or Rab7 levels (Figure 6B,C). Since Rab7 is not only a structural marker for specific vesicles, but is also in charge of proper vesicle transport, we investigated the activation state and whether inhibition of Rab7 alters the toxin-induced effect on the lysosome. Since TcdB inserts into the membrane of late endosomes, interference with Rab7 cannot be excluded. To test whether Rab7 is directly causative for observed TcdB effects on LC3B and cathepsin D, we applied the Rab7 inhibitor CID1067700. We found that CID1067700 alone had no effect on cathepsin D or LC3B, nor did it alter TcdB-induced changes (Figure 6D). Results showed no significant alteration of TcdB-induced decrease in cathepsin D or increase in LC3B-II (Figure 6E,F). To show that this effect is not a cell culture artefact, we additionally tested murine colon organoids. The increase in Rab7 was also observed in these organoids, representing primary cells and a more physiological model (Figure 6G). As shown by immunofluorescent staining of active, GTP-bound Rab7, the increase in Rab7, as shown by Western blots, correlated with an increase in Rab7-GTP in HEp-2 cells (Figure 6H). Active Rab7 increased progressively during TcdB NXN treatment, with its effects on the endolysosomal system indicated by EEA1 aggregation. This was verified by a GST-RILP-based pull-down assay.

Immunofluorescent analysis. Positive and negative controls were achieved by spiking cell lysates of untreated cells with non-hydrolyzable GTPγS or Mg^2+^-chelator EDTA, respectively. Indeed, GST-RILP-based pull down of Rab7-GTP yielded a higher signal in the TcdB NXN-treated compared to the untreated sample, indicating that TcdB NXN increased abundance of active Rab7 (Figure 6I). However, input samples also showed the general increase in Rab7 levels upon TcdB NXN exposure. TcdB NXN-mediated upregulation of Rab7, and therefore correlated with its activation and functional involvement in vesicular trafficking. Bafilomycin served again as the positive control, as impaired vesicle acidification triggers compensatory Rab7 activation [29]. Accordingly, bafilomycin-treated samples exhibited elevated levels of total Rab7 as well as Rab7-GTP. In accordance with the data shown above, cells treated with non-cleavable TcdB NXN H653A also appeared like untreated controls regarding total and active Rab7. Increased Rab7 activity was also observed at a 10-fold lower concentration of TcdB NXN, i.e., 100 ng/mL, in the absence of detectable early cell death (Figure S6).

### 2.7. Lysosomal Dysfunction Is a Common Effect of Many AB Toxins That Escape Endosomes

**Figure 7:**
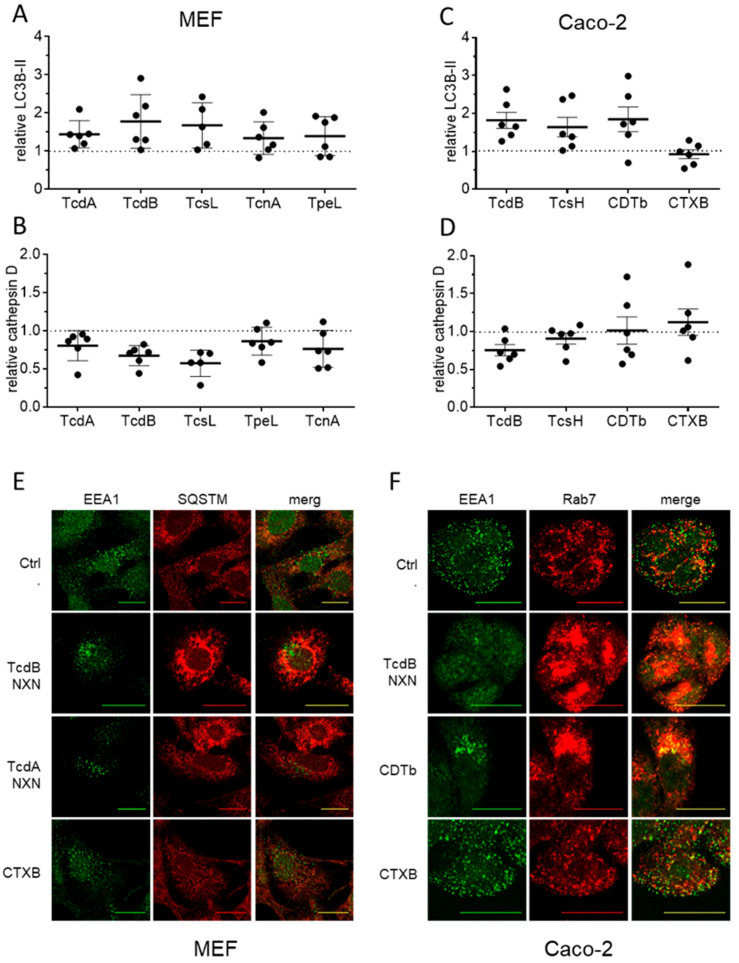
Interference with the endolysosomal pathway is a common feature of many AB toxins. (**A**,**B**) Densitometric evaluation of LC3B-II and cathepsin D immunoblots relative to β-actin. Mouse embryonic fibroblasts (MEF) were found to be sensitive to all large glucosyltransferases except TcsH. Graphs show data from six independent experiments (mean ± SEM). (**C**,**D**) Densitometric evaluation of LC3B-II and cathepsin D immunoblots relative to β-actin from Caco-2 cells, which are more sensitive to TcsH, and the B-subunits of the binary toxins CDT and CTX. TcdB served as a positive control and retrograde transported CTXB served as a negative control for change in LC3B-II and cathepsin D (n = 6, mean ± SEM). (**E**,**F**) Immunofluorescence pictures show the effect of all toxins tested in A–D for early change in early endosomes (EEA1, green) and amphisomes (SQSTM/Rab7, red). Shown are representative images from three independent experiments.

Many AB toxins employ a similar mechanism to deliver their active domains to the cytosol. The general mechanism of endosomal escape implies that the lysosomal impairment described here is not unique to TcdB. It ought to be a common consequence of endosomal escape by AB toxins. We therefore tested all large clostridial glucosyltransferases, i.e., TcdA and TcdB from C. difficile, TcsH and TcsL from Paeniclostridium sordellii, TpeL from C. perfringens and Tcn alpha (TcnA) from C. novyi as well as the binary toxin CDT from C. difficile in appropriate cell culture assays. To avoid any bias, we here used the wildtype form of all toxins to assess lysosomal dysfunction utilizing the established marker proteins cathepsin D and LC3B-II. We found mouse embryonic fibroblasts (MEFs) sufficiently sensitive to all large glucosyltransferases, except for TcsH (Figure 7A). After 6 h of treatment with 1 µg/mL TcdA, TcdB, TcsL, TpeL and TcnA, all toxins induced a significant increase in LC3B-II as the most sensitive marker. Accordingly, the decline in cathepsin D was also significant for all tested toxins following 24 h of treatment (Figure 7B). For experiments with TcsH, the binary toxin CDT from C. difficile, and cholera toxin from *V. cholerae* (CTX), we chose the human colon carcinoma cell line Caco-2. This intestinal epithelial cell line is an acknowledged model for endo- and transcytosis of CTX [30,31]. Like TcdB, TcsH induced an increase in LC3B-II and a decrease in cathepsin D after 6 h and 24 h, respectively (Figure 7C,D). However, even in Caco-2 cells the effect of TcsH was only weak, although borderline significant. Like single-chain toxins, oligomers of the pore-forming subunit of the C. difficile binary toxin CDTb also induced an increase in LC3B-II signal and reduction in cathepsin D level in Caco2 cells. 

The well-known CDTb-mediated endosomal pore formation validated the supposed mechanism of membrane integration as a reason for lysosomal dysfunction induced by multi-domain toxins. To show that the effect is specific for toxins that escape early to late endosomes to deliver their active domains to the cytosol, we additionally applied the B-subunit of cholera toxin (CTXB). Unlike the B-subunit of the binary toxin CDT (CDTb), CTXB, which enters cells by retrograde transport via the endoplasmic reticulum, did not significantly affect LC3B-II or cathepsin D in Caco-2 cells (Figure 7C,D). CTXB therefore serves as another appropriate negative control excluding mere endocytosis-related processes as reason for lysosomal dysfunction.

Staining for EEA1 and SQSTM1 revealed that MEF cells responded to TcdB NXN exposure with endosomal aggregation and accumulation of amphisomes, consistent with the phenotype observed in HEp-2 cells (Figure 7E, compare Figure 2A). This was also true for TcdA NXN. Retrograde-transported CTXB served as a negative control and did not affect EEA1 distribution or SQSTM1 signal intensity accordingly. To evaluate this effect on endosomal and amphisomal vesicles in Caco2 cells, we stained for EEA1 and Rab7 (Figure 7F). TcdB NXN served as a positive control and induced the characteristic changes in Rab7, i.e., increased abundance and perinuclear translocation, in Caco-2 cells, consistent with SQSTM1 in MEF cells. The same effect was observed after treatment with CDTb, but not with the negative control CTXB, validating that CDTb affects lysosomes and induces an increase in autophagic vesicles as shown in Western blot analyses and immunofluorescence staining.

### 2.8. Endosomal Escape Is Not Necessarily Connected with Change in Lysosome Function and Autophagic Flux

**Figure 8:**
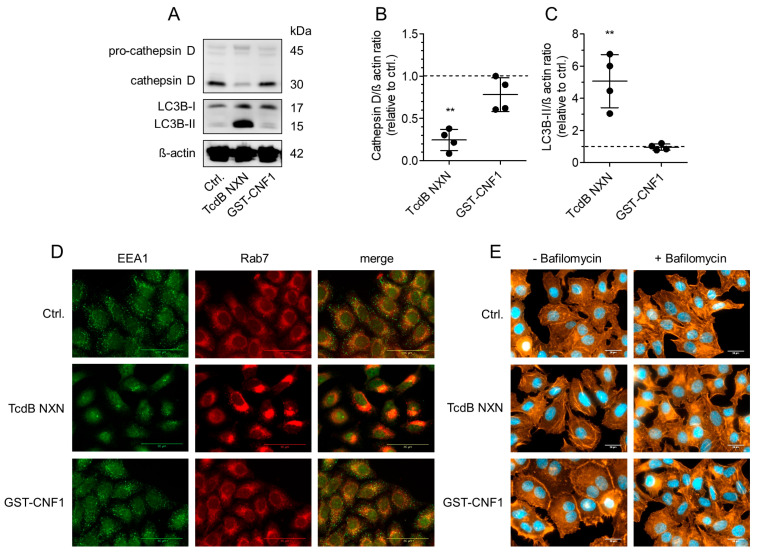
Cytotoxic necrotizing factor 1 does not affect the endolysosomal and autophagic pathway. (**A**) Immunoblots of HEp-2 cells show that in contrast to (600 µg/mL) TcdB NXN, the cytotoxic necrotizing factor 1 (GST-CNF1, 1 µg/mL) does not affect cathepsin D and LC3B-II compared to untreated controls. (**B**,**C**) Densitometrical evaluation of immunoblots from four separate experiments shows significant changes of cathepsin D and LC3B-II for TcdB but not for GST-CNF1. Shown are mean values ± SE, n = 4. (**D**) A total of 1 µg/mL GST-CNF1 also did not affect EEA1 and Rab7 localization after 24 h treatment in HEp-2 cells. TcdB NXN was applied as a positive control. (**E**) Rhodamine-phalloidin staining of the actin cytoskeleton (red) and; Hoechst staining of nuclei (blue) was performed to visualize the pathological effect of GST-CNF1, i.e., bi-nucleation and increase in cortical actin cytoskeleton after 24 h. Addition of 10 nM bafilomycin A1 prevented GST-CNF1 (1 µg/mL) effects and proved pH-dependent endosomal egress of CNF1. TcdB NXN (1 µg/mL) showed no major changes in actin cytoskeleton and nucleus.). ** indicate significances of a *p*-value < 0.01.

Multi-domain AB-toxins with distinct receptor binding and enzymatically active domains integrate into endosomal membranes to translocate the pathogenic enzyme domain into the cytosol. This process depends on acidification of the endosomal lumen. Smaller and less complex AB-toxins like cytotoxic necrotizing factor 1 (CNF1) from *E. coli* also translocate from the endosome to the cytosolic compartment in a pH-dependent manner [32]. In contrast to LCGTs, the C-terminal subdomain of CNF1 harbors deamidase activity and is also cleaved off upon translocation to the cytosol [33]. Therefore, we also tested this inverse AB-toxin for its capability to interfere with endolysosomal transport and autophagic flux. Western blots from Hep-2 cell lysates revealed that CNF1 did not significantly alter cathepsin D or LC3B level compared to untreated control cells after 24 h of incubation (Figure 8A–C). This was also true for 6 h of treatment with CNF1. Immunofluorescence staining revealed lack of effect on early endosomes (indicated by EEA1 staining) and amphisomal vesicles (indicated by Rab7 staining) (Figure 8D). Whereas TcdB NXN induced a reduction in peripheral EEA1 and perinuclear accumulation of Rab7, GST-CNF1-treated HEp-2 cells showed distribution and abundance of these two marker proteins comparable to untreated controls. To validate the entry and cytopathic effect of GST-CNF1 into HEp-2 cells, rhodamine phalloidin staining was performed along with DAPI-staining to show a typical CNF1 phenotype, i.e., increase in actin cytoskeleton and multi-nucleation, respectively. GST-CNF1 induced a strong increase in cortical F-actin and a high percentage of bi-nucleated cells after treatment of HEp2-cells for 24 h (Figure 8E). This effect was abolished by bafilomycin A1, validating pH-dependent endosomal egress of GST-CNF1. Thus, unlike large multi-domain toxins, smaller single-chain AB-toxins with an inverse order of domain architecture likely do not induce membrane perforation that sustainably interferes with endosomal ion homeostasis.

## 3. Discussion

As previously described, TcdB affects lysosomes, resulting in an overall decreased degradation capacity of cells, accompanied by a delayed elimination of endolysosomal cargo [8]. Applying an appropriate functional marker for lysosomes (e. g. cathepsin D) and autophagy (e. g. LC3B), we here described how lysosomal dysfunction is a progressive effect over the observed period of 24 h. Even a concentration of 1 ng/mL TcdB NXN was sufficient to induce endosomal aggregation of EEA1 puncta and Rab7 signal increase after 24 h of exposure, indicating non-functional endolysosomes and amphisomes, respectively (Figure 4A). These findings underline that endocytic uptake of toxin in realistic pathophysiological concentrations, which would mean up to 300 ng/mL stool according to real-time cell analysis [34], bears an additional pathogenic quality of specific toxins independent of their enzymatic activity. Of course, in case of large clostridial glucosyltransferases, Rho/Ras-glucosylation dominates all other effects, eventually leading to apoptotic cell death. It is, however, unclear how induction of lysosomal dysfunction and disturbed autophagy as described here affect cells in the early phase of intoxication and how it modulates pro-apoptotic and apoptotic processes.

Membrane integration of TcdB in combination with release of the translocated glucosyltransferase domain causes minor damage to endosomal membranes, thereby recruiting the ESCRT-III membrane repair machinery (Figure 6C). While the toxin-mediated damage is not sufficient to evoke galectin-3/8 response [9], it may impair endosomal homeostasis to hinder lysosomal function upon endolysosomal fusion. The ESCRT-III machinery is transiently recruited to sites of acute damage [15]. CHMP4B accumulation at late endosomes indicates TcdB NXN-induced ESCRT-III recruitment, which persists up to 24 h (Figure 6D), indicating that at least some of the toxins remain integrated into the endosome membrane and trigger sustained membrane repair. We hypothesize that TcdB NXN-induced perforation of internalizing endosomes likely exceeded the capacity or capability of the cell’s ESCRT-III membrane repair system leading to cumulative lysosomal damage.

As endosomes progress and fuse with lysosomes, they are trafficked toward the microtubule-organizing center (MTOC) in the cell center [35]. Under physiological conditions, these lysosomal vesicles near the nucleus are highly acidic and do not possess early endosome marker proteins. In our study, the TcdB NXN-induced perinuclear aggregates displayed endosomal as well as lysosomal markers near the nucleus (EEA1 and cathepsin D), indicating impaired maturation of endolysosomal vesicles. Additionally, TcdB NXN-mediated endosomal aggregates did not exhibit Rab7 signal, indicating that they did not properly mature to late endosomes, or they lost Rab7 due to sequestering in other compartments. Formation of proto-lysosomes requires content condensation, which depends on sufficient acidification and luminal Ca2+ [36]. The alterations in endosomal and autophagosomal markers as well as the decrease in cathepsin D levels induced by TcdB reflect non-functional vesicles, which in turn might affect re-formation of functional lysosomes. It is generally acknowledged that integration of toxins into the endosomal membrane leads to membrane perforation. Barth and coworkers documented increased 86Rb^+^ release from preloaded CHO cells upon pH-induced integration of TcdB or TcsL into the plasma membrane, suggesting that toxin-induced membrane perforation functions as an ion channel as shown by increased current in lipid bilayer experiments [37]. Whether membrane perforation was transient or persistent is not known. According to Barth and coworkers, lipid bilayer experiments reveal pores with small lifetimes [37]. Our results suggest similar membrane leaks, which lead to the impairment of endosomal homeostasis and lysosomal metabolism. Nothing is known about the nature of such a leak in the endosome membrane. It could just as easily be that the toxin allosterically modulates ion channels, thereby indirectly affecting ion homeostasis. As shown by bafilomycin experiments, inhibition of vATPase induces the same effects as TcdB regarding cathepsin D, LC3B, Rab7, and SQSTM1. Of note, Chan and coworkers reported downregulation of vATPase by TcdB, leading to lysosome dysfunction and autophagosomal death in macrophages, speaking in favor of an indirect effect on ion homeostasis [38]. However, CHMP4B accumulation as a marker for membrane damage, a requirement of GTD release, the fact that the effect is not restricted to TcdB, and the positive control with CDTb as a well-known pore-forming toxin are strong arguments that lead us to believe that membrane leakage is causative for these effects. It is common sense that large clostridial glucosyltransferases function as monomers. To date, there is no indication that refers to oligomerization of these large toxins for membrane insertion, including our present data. The precise mechanism is yet to be determined. Whether vATPase or ion channels/transporters are additionally affected by an alteration in ion homeostasis (or membrane potential/conductance) is an interesting question that must be investigated in the future.

**Figure 9:**
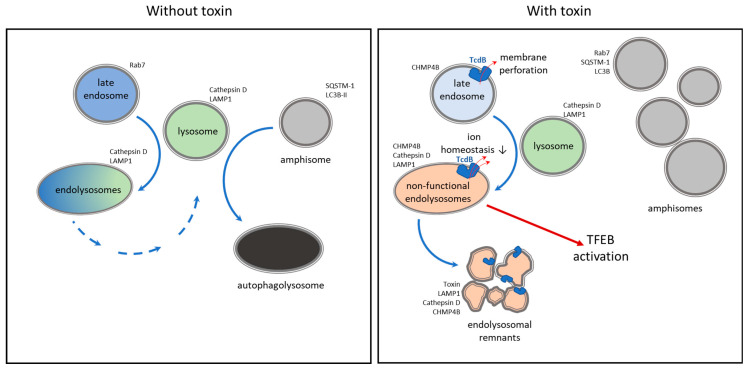
Schematic of lysosomes centrally involved in processing late endosomes and amphisomes/autophagosomes with specific marker proteins for various vesicular structures (**left panel**). pH-dependent insertion of TcdB into the membrane interferes with the degradation process after fusion with lysosomes and causes accumulation of endolysosomal remnants as well as secondary accumulation of amphisomes and activation of TFEB for compensatory reconstitution of lysosomes (**right panel**).

Immunofluorescence analyses of TcdB NXN-treated cells reveal two distinct phenomena: (i) large aggregates which are positive in EEA-1, LAMP1/cathepsin D, CHMP4B and TcdB staining, and (ii) a perinuclear concentration of vesicles positive for SQSTM, Rab7, and LC3B. Based on these characteristic marker proteins, the first mentioned aggregates likely resemble damaged and non-mature endolysosomes still containing TcdB NXN. This implies a direct toxin effect. The second mentioned increase in the pool of vesicles was also observed in almost all toxin-treated cells in TEM experiments. These structures were less prominent in the cell periphery, and high magnification revealed a lack of autophagosomal characteristics such as a double layer of membrane and electron-dense vesicular content. Thus, these structures resemble autolysosomes or amphisomes, which are described as non-mature endosomal hybrid vesicles (amphisomes) containing non-processed waste [39]. Amphisomes accumulate in the absence of the HOPS complex due to a lack of fusion with functional lysosomes [40], suggesting that TcdB-induced endosomal disruption may inhibit their lysosomal fusion. However, a clear description and characterization of these abnormal structures is not possible by mere immunofluorescence staining or morphological description. All toxin-induced changes in lysosome-associated processes are graphically summarized in Figure 9.

In general, Rab7 is an acknowledged marker for late endosomes, and regulates fusion of endosomes and autophagosomes with lysosomes [18]. Rab7 activity also accounts for perinuclear localization, as Rab7-RILP-mediated transport along the microtubule is minus-end directed toward the MTOC near the nucleus [35]. Impaired vesicle acidification has been reported to trigger compensatory Rab7 activation [29]. Thus, in our study, we assume that TcdB NXN-induced increase in total and active Rab7 is a compensatory mechanism for impaired vesicle acidification. This is supported by bafilomycin A1 experiments, showing the same effect of Rab7 activation, based on inhibition of endosome acidification. Interestingly, immunofluorescence staining showed complete co-localization of Rab7 and SQSTM1, reflecting an increase in maturing autophagosomes, rather than late endosomes. To test whether TcdB induces lysosomal dysfunction by impaired formation of late endosomes, we applied the Rab7 inhibitor CID1067700. Although Rab7 was largely inhibited, we did not observe any effects on lysosomes and autophagosomes. In addition, CID1067700 did not prevent TcdB-mediated effects. From this data, we conclude that Rab7 is unlikely to serve as a key regulator here. Thus, we interpret our data in the way that increased Rab7 is a second-tier mechanism and serves in our experiments as a marker for compensatory increase in maturing autophagosomes.

We here reported the activation of transcription factor EB (TFEB) by TcdB. TFEB translocates into the nucleus to increase gene expression for synthesis of lysosomes and autophagosomes in a Ca^2+^-dependent manner [41]. TFEB translocated to the nucleus upon TcdB NXN-induced lysosomal impairment to mediate lysosomal biogenesis (Figure 8C). However, following 24 h of TcdB NXN exposure, TFEB levels were still strongly decreased. As described by Xu and coworkers, compromised transcriptional activity potentially led to a reduction in TFEB protein levels [42]. Dephosphorylation of TFEB is triggered by Ca^2+^-release from endolysosomes either by membrane damage (mimicked by LLOMe) or by calcium channels, such as the transient receptor potential cation channel, mucolipin subfamily (TRPML) or L-type voltage-gated Ca^2+^ channel. Of note, inhibition of the L-type voltage-gated Ca^2+^ channel by nifedipine prevented TcdB-induced early cell death, validating toxin-induced Ca^2+^ signaling [9,19]. The mechanism for this is unknown. Toxin-induced membrane perforation may alter membrane potential, thereby activation of voltage gated ion channels. However, toxin-built leaks may also directly allow additional ion efflux. Increased cytosolic Ca^2+^ activates calcineurin to dephosphorylate TFEB as a prerequisite for nuclear translocation [41]. Notably, although inhibiting early cell death, nifedipine did not alter toxin-induced lysosomal dysfunction nor TFEB translocation into the nucleus (Figure S5). This might indicate another and indirect regulatory mechanism for toxin-induced TFEB activation and is currently under investigation.

As exemplarily shown by application of glucosyltransferase-deficient TcdB (TcdB NXN), pH-dependent integration into the endosomal membrane affects lysosomes and autophagic flux independent of Rho glucosylation. Consequently, a general mechanism is likely to apply to all glucosyltransferases. We validated this mechanism for all LCGTs as well as for the oligomer-forming B-subunit of the binary toxin CDT from C. difficile, which also inserts into the endosomal membrane after vesicle acidification. It remains to be clarified why smaller AB-toxins such as CNF1 do not interfere with lysosome function. As mentioned above, similar to LCGTs, CNF1 exits the endosome in a pH-dependent manner and the catalytically active deamidase domain is cleaved off [33]. Our results indicate that CNF1 does not perforate the endosomal membrane in a way that significantly and persistently disturbs ion homeostasis.

The impact of dysregulation of endolysosomal transport and autophagic flux on pathogenesis in C. difficile infection is not clear yet and has to be investigated in the following. Our experiments were performed in an established in vitro model which bears the advantage of reproducibility, manipulation by transfection, and microscopical imaging, but lacks the validity of an in vivo system. Inclusion of organoids is only a first step for transferring findings to organ pathology and in vivo situations. Using this artificial system, we were able to characterize and dissect the effect on lysosomes from dominant pathogenic toxin effects based on Rho inhibition. Notably, no clinically relevant C. difficile strain producing glucosyltransferase deficient toxin has been described so far. However, it must be emphasized that current vaccines include detoxified TcdB (D286/297N mutant) and are well tolerated [43,44]. This speaks against an intrinsic and additional pathogenic effect, but does not exclude modulatory effects, for example, on the immune system. A broader approach including transcriptomics might help to understand this effect.

In summary, our data confirm that many AB structure type toxins affect target cells simply by perforating the endosomal membrane, independent of their main pathogenic effect. Perforating the endosomal membrane results in lysosomal dysfunction and, depending on the dose and uptake characteristics, may even lead to cell death.

## 4. Conclusions

Many bacterial toxins integrate into the endosome membrane to access cytosol, thereby potentially affecting membrane integrity. The lysosomal dysfunction induced by TcdB is representative for all large clostridial glucosyltransferases and seems to be a common mechanism for many but not for all toxins. The impact of this event in pathogenesis is not clear yet. A contribution to pro-inflammatory processes, malfunctioning of antigen presenting cells, or senescence can be hypothesized to modulate or contribute to pathogenic enzyme activities of these toxins.

## 5. Materials and Methods

### 5.1. Antibodies Used

Primary antibodies: Antibodies anti-CHMP4B (IF; #136831-AP) were obtained from ProteinTech, Planegg-Martinsried, German); anti-Cathepsin D (IF/WB; #6985), anti-EEA1 (IF;2411), anti-LC3B (IF/WB; 3868), anti-SQSTM1 (IF/WB; #88588), anti-Rab7 (IF/WB; 95746), and anti-GAPDH (WB;2118) were all from Cell Signaling Technology, Leiden, The Netherlands; anti- beta-actin (WB; #A5441) was obtained from Sigma Aldrich/Merck, Darmstadt, Germany; anti-Rab7-GTP (IF; #26923) was obtained from NewEast Biosciences, Glenmoore, PA, U.S.A..; anti-LAMP1 (IF; #SC17768) was obtained from Santa Cruz, Heidelberg, Germany; and anti-TcdB D286/288N (IF/WB) was made in-house [13].

Secondary anti-IgG antibodies: Anti-mouse-AF594 (#A-11005), anti-mouse-AF488 (#A-11001), anti-rabbit-AF594 (#A-11012), and anti-rabbit-AF488 (#11008) were all from Invitrogen, Scherte, Germany.

Yersinia mollaretii for cloning of YGT was obtained from the German collection of microorganisms and cell culture, DSMZ, (Catalog number: DSM 18520). The coding sequence for TpEL (strain C. perfringens NCTC3812) was generated in three parts (bp 1–1266; 1267–3495; 3496–5337) ligated into pHIS1522 to code for the full-length protein with C-terminal 6 × His tag and sequenced (Eurofins, Cologne, Germany).

Chemicals: CID1067700 (ML282) was from MedChem Express/Merck, Millipore, Darmstadt, Germany, #HY-13452; Phalloidin-Rhodamine (#R415) was from Molecular Probes/Thermo Fisher Scientific, Schwerte, Germany. Nifedipin was from Sigma/Merck, Darmstadt, Germany (#N7634-5G). All standard chemicals were of highest available purity.

### 5.2. Purification of Native Clostridial Glycosyltransferases

All native toxins were purified from 2 L supernatant of clostridial cultures by 70% ammonium sulfate precipitation for 2 h at 4 °C, resuspension and dialysis against 20 mM Tris-HCL pH 7.4 and 50 mM NaCl, and subsequent ion-exchange chromatography as described by Krivan and Wilkins [45]. Eluted fractions (40 × 1 mL) were analyzed by 7.5% SDS-PAGE and fractions with the highest concentrations of specific toxins were separately pooled, concentrated by Amicons with a 100 kDa cut-off membrane and dialyzed against 20 mM Tris-HCl pH 7.4 and 50 mM NaCl. Aliquots of 50 µL were stored at −80 °C until use.

### 5.3. Expression of Recombinant Large Clostridial Glucosyltransferases

All recombinant large glycosyltransferases (TcdA and TcdB from C. difficile strain VPI10463; TcsL from P. sordellii strain 6018; TpEL from C. perfringens strain NCTC3812; TcsH from C. sordellii strain VPI9048; Tcn alpha from C. novyi strain ATCC 19402) were expressed with C-terminal 6 × His-tag using the Bacillus megaterium expression system as initially described for TcdA [46] or TcdB [24]. Coding sequences of toxin genes were cloned via BsrGI and KpnI into pHIS1522, except TcdA, which was cloned via BamHI into a modified pWH1520 vector. Site-directed mutagenesis was performed using the Quick Change XL site-directed mutagenesis kit from Agilent, Waldbronn, Germany. Toxins were expressed overnight at 28 °C. Bacteria were harvested by centrifugation and resuspended in 20 mL LEW buffer for Ni-IDA affinity chromatography (Macherey-Nagel) and lysed by sonification on ice. Cell lysates were centrifugated and the supernatant was sterile filtered (0.45 µm pore size) and subjected to Ni-IDA columns for purification of His-tagged proteins. The eluted toxins were further purified by size exclusion chromatography (Superose 6, Cytiva) with PBS as the running buffer. Purified toxins were stored in appropriate aliquots at −80 °C until use.

### 5.4. Expression of CDTb, GST-CNF1 and Other GST-Fusion Proteins

CDTb (from C. difficile strain R20291) was cloned into a pQE30 vector with an N-terminal 6 × His tag and expressed in *E. coli* and purified as described by Beer and coworkers [47,48]. In brief, the protein was purified via Protino Ni-IDA resin (Macherey-Nagel, Düren, Germany), activated by trypsin (0.2 µg/µg CDTb protein; Sigma/Merck, Darmstadt, Germany) for 30 min at room temperature. Trypsin was then inhibited by addition of benzamidine, and samples were dialyzed against PBS. All GST-fusion proteins were expressed in *E. coli* under standard conditions. CDTa and CDTaN-YGT were cloned into a pGEX 2TGL vector, expressed in *E. coli*, and purified as described by Beer and coworkers [47]. CDTaN-YGT consists of the N-terminal amino acids 44-266 of CDTa fused to amino acids 1-521 of the glucosyltransferase from Yersinia mollaretii [17]. GST-CDTaN fusion proteins were cleaved by thrombin to obtain the tag-free proteins [16]. GST-CNF1 was produced as previously described by [32,49]. For Rab7 pull-down assays, the coding sequence of the Rab7 interacting domain of RILP (amino acids 240-320) [19,20] was amplified from cDNA generated from Hep-2 cell total mRNA using Q5 polymerase. The sequence was cloned into a pGEX2TGL vector via compatible BamHI/BglII overhang and EcoRI and expressed as GST-fusion protein (GST-RILP 240-320).

### 5.5. Cell Culture and Transfection of Hep-2 Cells

The human epithelial-like cell line Hep-2 was cultured in minimum essential medium (MEM) with 10% FCS and penicillin/streptomycin under standard conditions. Cells were subcultured twice a week. Transfection of cells was performed on cover slips in 24 wells at 30% confluency. Therefore, cells were transfected with pcDNA3.1-EGFP or pcDNA3.1-EGFP-TcdB 543-800 coding for EGFP alone or the EGFP/cysteine protease domain fusion protein. Cells were transfected using jetPRIME^®^ transfection reagent as per the standard protocol provided by the supplier (Polyplus-Sartorius, Göttingen, Germany). Cells were incubated for 24 h and processed as described under immunofluorescence in the following.

### 5.6. Rab7GTP Pull-Down Assay

HEp-2 cells were seeded on 6-well cell culture dishes at a density of 52,000 cm^−2^. The following day, cells were treated with the specified toxin for the indicated incubation time. After treatment, the culture medium was removed, cells on culture dishes were put on ice and incubated with 0.5 mL/well ice-cold fishing buffer (50 mM Tris-HCl, pH 7.4, 100 mM NaCl, 2 mM MgCl2, 10% [*v*/*v*] glycerol, 1% [*v*/*v*] NP40, 0.5 mg/mL BSA) for 5 min. The cell lysates of two wells were combined in a clean tube and centrifuged at 10,000× *g* for 10 min at 4 °C. A total of 800 µL of the supernatant was then transferred to 40 µLof bead slurry of previously prepared GST-RILP fusion protein (app. 20 µg) immobilized to GSH-sepharose. The samples were rotated for 30 min at 4 °C. The beads were subsequently washed twice in fishing buffer. Each bead pellet was then re-suspended in 40 µL 2.5 × Laemmli buffer. The samples were incubated at 95 °C for 3 min, briefly centrifuged and stored at −20 °C for subsequent SDS-PAGE.

### 5.7. Immunofluorescence

HEp-2 cells were seeded on cover slips at a density of 52,000 cm^−2^ and treated with the specified toxin for the indicated incubation period the following day. Cells were then fixed in 4% paraformaldehyde in PBS or methanol (−20 °C) for 20 min at 4 °C and subsequently washed three times with room temperature PBS. Cells were further permeabilized with 0.3% Triton X-100 in PBS at room temperature for 5 min and washed three times with room temperature PBS. Thereafter, samples were blocked with 5% bovine serum albumin in PBS for 1.5 h. The samples were incubated with primary antibodies overnight at 4 °C. Subsequently, the samples were washed three times with PBS at room temperature. The appropriate secondary antibody was incubated with the samples overnight at 4 °C. All antibodies were diluted 1:100 in PBS containing 2.5% (*w*/*v*) bovine serum albumin. After washing with PBS followed by one washing step with H_2_O at room temperature, cells were embedded in mounting medium and analyzed by confocal laser scanning microscopy using the 60 × oil immersion objective of a Leica Inverted-3 microscope. Fluorescent dyes were excited in sequence at a wavelength of 475 nm and 555 nm, respectively. The samples were documented by Zeiss microscope Axio Observer 3/5/7 KMAT and software Zen version 3.1. All images are representative for at least three independent biological replicates each with three experiments in parallel (technical replicates) unless otherwise stated. Image analysis was performed using ImageJ software version 1.54g.

### 5.8. Western Blot Analyses

Cell lysates prepared in Laemmli buffer were subjected to SDS-PAGE using 7.5–15% polyacrylamide gels depending on the molecular weight of the specific target protein. Resolved proteins were transferred onto nitrocellulose membranes by semi-dry blotting. Membranes were rinsed with Tris-buffered saline containing 0.2% Tween-20 (TBS-T), then stained with Ponceau S to verify protein transfer. Once destained in TBS-T for 5 min, membranes were blocked in 5% skimmed milk powder in TBS-T for 1 h at room temperature. Following a brief wash with TBS-T, membranes were incubated overnight at 4 °C with primary antibodies.

All antibodies were diluted 1:1000 in TBS-T except anti-LC3B, which was diluted 1:1000 in TBS-T containing 5% (*w*/*v*) bovine serum albumin. Membranes were subsequently washed and incubated with the appropriate secondary antibody for 1 h at room temperature. Horseradish peroxidase (HRP)-conjugated goat anti-rabbit, anti-mouse or anti-rat (all from Rockland) were each diluted 1:5000 in TBS-T. After extensive washing, signals were visualized using ECL Western Blotting substrate and imaged with the Intas™ ChemoStar Imager (Intas Science Imaging Instruments, Göttingen, Germany). Signal intensities were quantified by densiometric analysis using ImageJ software (version 1.54g).

### 5.9. Transmission Electron Microscopy

Cells were seeded in 72 cm^2^ culture flasks to reach 50–70% confluency the day after seeding. Cells were treated as indicated and incubated for 24 h. Culture medium was carefully removed, and cells were fixed in culture flasks for 30 min in 150 mM HEPES, pH 7.35, containing 1.5% formaldehyde and 1.5% glutaraldehyde. Afterwards, cells were gently scraped off in 1 mL fixation solution and transferred into an Eppendorf cup, sedimented at 100× *g* for 5 min. The supernatant was carefully aspirated and the resulting cell pellet was immersed in 2% agarose (40 °C). After immobilization in agarose, samples were incubated for 2 hr in an aqueous solution of 1% OsO4 containing 1.5% hexacyanoferrate II, washed in water and stored in 1% aqueous uranyl acetate overnight at 4 °C. Samples were washed in water and dehydrated in acetone and finally embedded in Epon. Ultra-thin sections (60 nm) were mounted on formvar-coated copper grids, post-stained with uranyl acetate and lead citrate and observed in a Morgagni TEM (FEI) at 80 kV. Images from two separate grids with non-consecutive ultra-thin slices per sample (two biological replicates) were recorded using a side-mounted 2K-Veleta CCD camera.

### 5.10. Statistics

All data were analyzed with GraphPad Prism 6.0. Graphs show mean values ± standard error of mean (SEM), if not indicated otherwise. Significance was tested with a student’s *T*-test and indicated as follows: n. s., non-significant; * *p*-value < 0.05; ** *p*-value < 0.01; and *** *p*-value < 0.001.

## Data Availability

The original contributions presented in this study are included in the article/Supplementary Material. Further inquiries can be directed to the corresponding author.

## Supplementary Materials

The following supporting information can be downloaded at https://www.mdpi.com/article/10.3390/toxins18040186/s1: Figure S1: Cathepsin D activity in Hep-2 cells treated with cleavable and non-cleavable mutant form of TcdB NXN; Figure S2: Immunofluorescence staining of Rab7 and cathepsin D in Hep-2 cells treated with TcdB NXN; Figure S3: Immunofluorescence staining of TcdB NXN and non-cleavable TcdB NXN H653A in colocalization with LAMP1. Figure S4: Effect of calcium channel inhibitor nifedipine on TcdB NXN-induced CHMP4B accumulation at endosomes; Figure S5: Immunofluorescence staining of TFEB translocation into nucleus after treatment of hEp-2 cells with TcdB NXN; Figure S6: Validation of GST-RILP pull-down assay for analyses of Rab7 activation at lower toxin concentration. Application of Rab7 inhibitor CID1067700.

## Abbreviations

The following abbreviations are used in this manuscript:

TcdA	*Clostridioides difficile* Toxin A
TcdB	*Clostridioides difficile* Toxin B
TcdB NXN	*Clostridioides difficile* Toxin B D286/288N, Glucosyltransferase-deficient Mutant
CDTb	*Clostridioides difficile* Transferase, B-subunit
CTXB	*Cholera-Toxin, B-Subunit*
TcsH	*Paeniclostridium sordellii* Hemorrhagic Toxin
TcsL	*Paeniclostridium sordellii* Lethal Toxin
TcnA	*Clostridium novyi* alpha-Toxin
TpEL	*Clostridium perfringens* Large Enterotoxin
LCGTs	Large Clostridial Glucosyltransferases
CDTaN-YGT	Fusin Protein of the N-terminal Adapter Domain of *Clostridioides difficile* Transferase, A-subunit with the Glucosyltransferase Domain of *Yersinia mollaretii* YG
