# Supplementary material for: Interference of Large Clostridial Glucosyltransferases with the Endolysosomal Pathway: Toxin-Induced Imbalance of Early Endosomes, Functional Lysosomes and Autophagosomes
Source: Toxins (Basel). 2026 Apr 15;18(4):186. doi: 10.3390/toxins18040186 (PMC13119598; doi:10.3390/toxins18040186)
Supplement: Supplementary file 1 [file toxins-18-00186-s001.zip › toxins-4206905-supplementary.pdf]

Supplementary figures

Fig. S1: Cathepsin D activity in Hep-2 cells treated with cleavable and autoproteolytic deficient mutant form of TcdB NXN

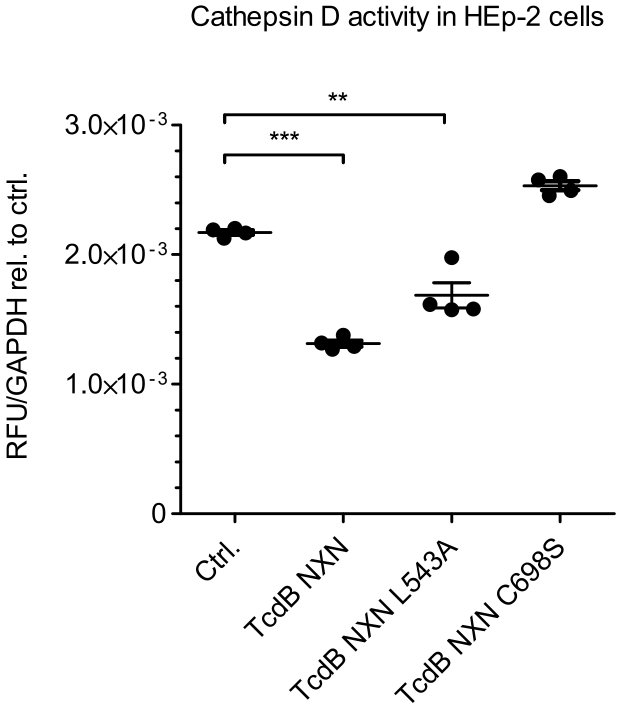

For measuring cathepsin D activity, HEp-2 cells were seeded in a 6-well format at a density of 40,000 cm^{-2} to generate at least 1 x 10^6 cells per sample. After treatment with the indicated toxin, the cells were rinsed with lukewarm PBS and covered in 0.5 ml Trypsin-EDTA. After 5 min incubation at 37 °C and 5 %CO_2, cells were rinsed off with PBS and pelleted at 800 x g for 5 min at room temperature. After resuspension in PBS, the cells were counted using a Neubauer counting chamber to adjust all samples to 10^6 cells per ml. Samples were processed according to fluorometric cathepsin D activity Kit instructions (abcam #ab65302) for cell culture. Therefore, cells were, lysed in lysis buffer and 50 µl of lysates were transferred to a black 96-well plate. Cathepsin D substrate was diluted in the appropriate reaction buffer and added to the samples according Kit instruction. Following 2 h incubation at 37 °C, samples were measured with PlateReader at Ex/Em 328/460 nm. In parallel to cathepsin D activity assay, 20 µl of cell lysate was used for western blot analyses to compare housekeeping protein GAPDH of samples for equal protein amounts. Shown are mean values ± SEM, n = 4. *** p-value <0.001, ** p-value >0.01. A significant reduction in cathepsin D activity relative to GAPDH content was observed in TcdB NXN and TCDB NXN L543A treated cells but not in autoproteolysis deficient TcdB NXN C698S treated cells, which is in line with cathepsin D abundance in samples according to western blot analyses.

Fig. S2: Immunofluorescence staining of Rab7 and cathepsin D in Hep-2 cells treated with TcdB NXN

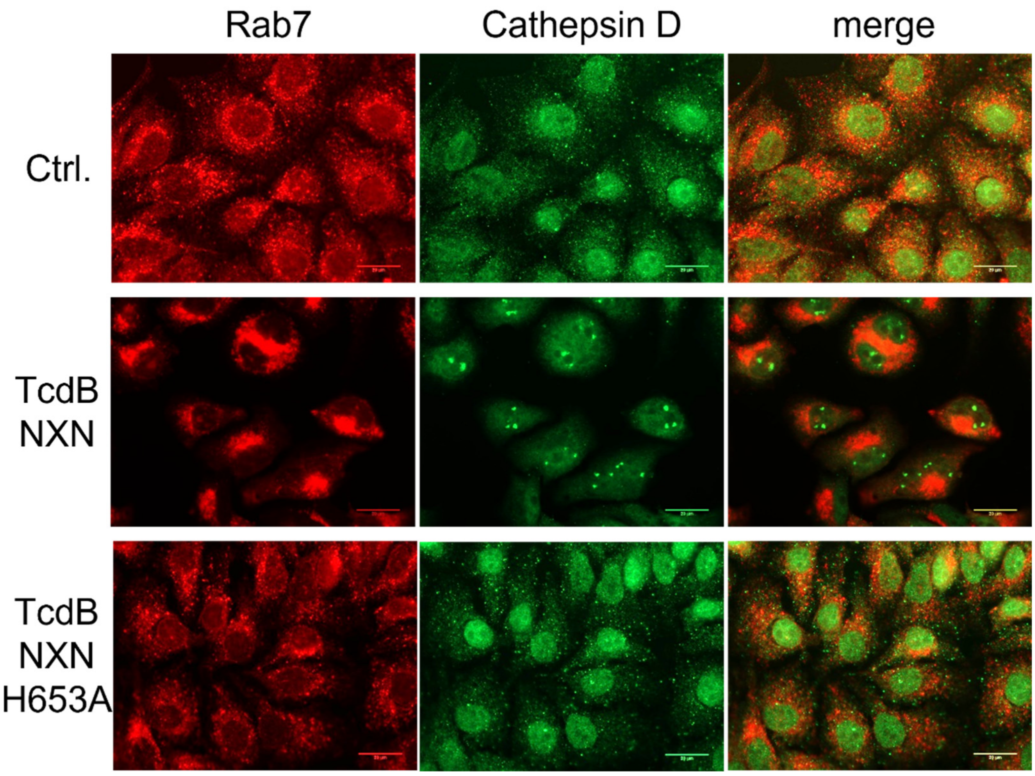

Immunofluorescence staining of Hep-2 cells after treatment with TcdB NXN or TcdB NXN H653A (1000 ng/ml, each). Cells were treated with toxins for 24 h. After treatment, cells were washed, fixed and stained for Rab7 (AF594, red) and for cathepsin D (AF488, green) as described in methods. TcdB NXN induced aggregates near the nucleus that were positive for cathepsin D but not for Rab7. Hep-2 cells treated with the non-cleavable muatant TcdB NXN H653A showed comparable distribution of Rab7 and cathepsin D like untreated control cells. Scale bars represent 20 µm.

Fig. S3: Immunofluorescence staining of TcdB NXN and non-cleavable TcdB NXN H653A in colocalization with LAMP1.

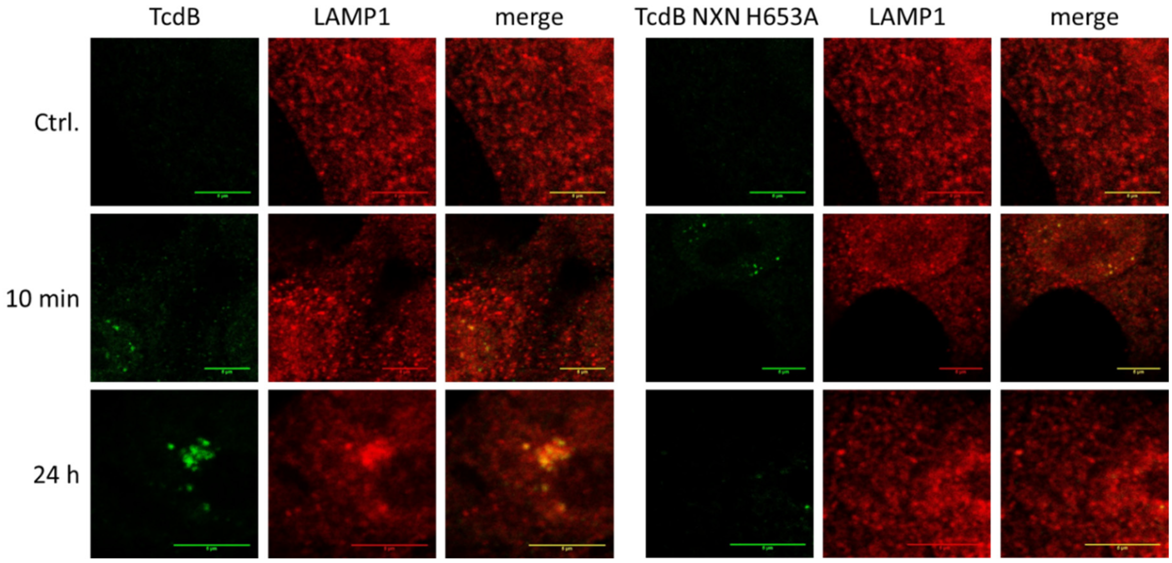

Immunofluorescence staining of Hep-2 cells after treatment with TcdB NXN or TcdB NXN H653A (1000 ng/ml, each). Cells were treated with toxins for 10 min or 24 h. After treatment for indicated times, cells were washed, fixed and stained with polyclonal rabbit anti-TcdB IgG/anti-rabbit AF488 (green) or mouse anti-LAMP1/anti-mouse AF594 (red) as described in methods. TcdB NXN and TcdB NXN H653A were detected in vesicular structures after 10 min that were also positive for LAMP1. Whereas TcdB NXN positive vesicles accumulated in the perinuclear region over 24 hours, TcdB NXN H653A was less than observed after 10 min, indicating lysosomal degradation of TcdB NXN H653A but not of TcdB NXN after endocytosis.

Scale bars represent 5 µm.

Fig. S4: Effect of calcium channel inhibitor nifedipine on TcdB NXN-induced CHMP4B accumulation at endosomes

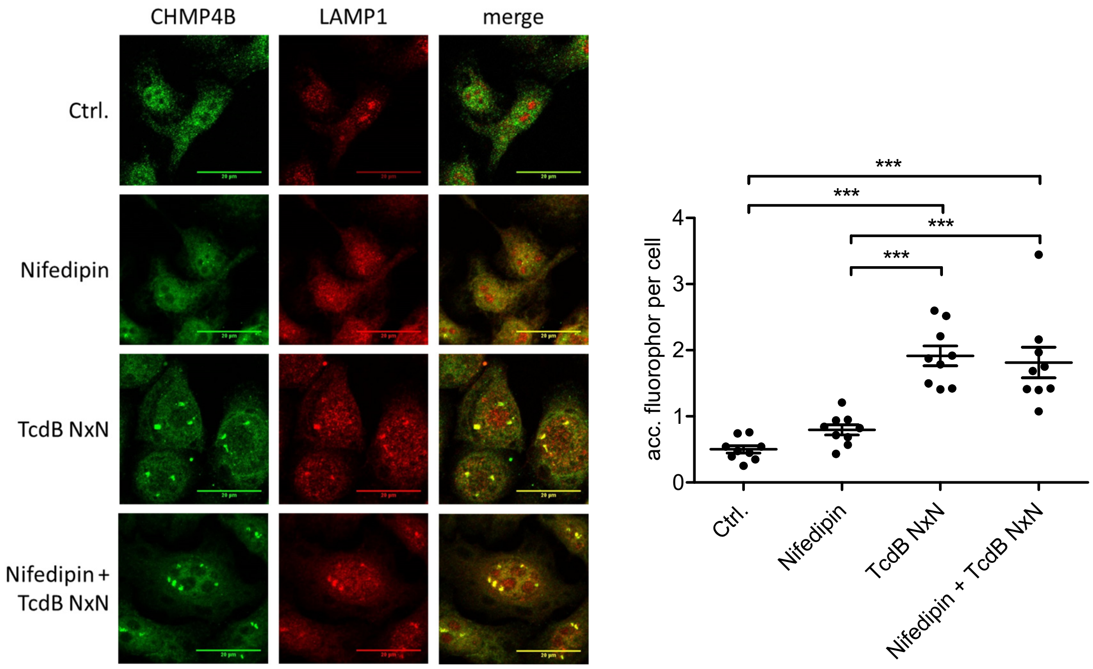

Inhibition of voltage-sensitive L-type Ca^{2+} channels by nifedipine does not affect TcdB NXN-induced CHMP4B accumulation at endolysosomes. Cells were treated with 1000 ng/ml of indicated toxins for 6 hours and stained for CHMP4B (AF488; green) and LAMP1 (AF594; red). TcdB NXN induced CHMP4B accumulation at late endosomes/endolysosomes that were also positive for LAMP1 (left panel). Treatment of cells with nifedipine did not abolish CHMP4B accumulation. Quantification of CHMP4B positive vesicles from eight independent samples revealed significant increase in number of CHMP4B accumulation compared to controls. Nifedipin did not significantly reduce number of CHMP4B vesicles in TcdB NXN treated samples. Scale bars represent 20 µm.

Fig. S5: Immunofluorescence staining of TFEB translocation into nucleus after treatment of hEp-2 cells with TcdB NXN

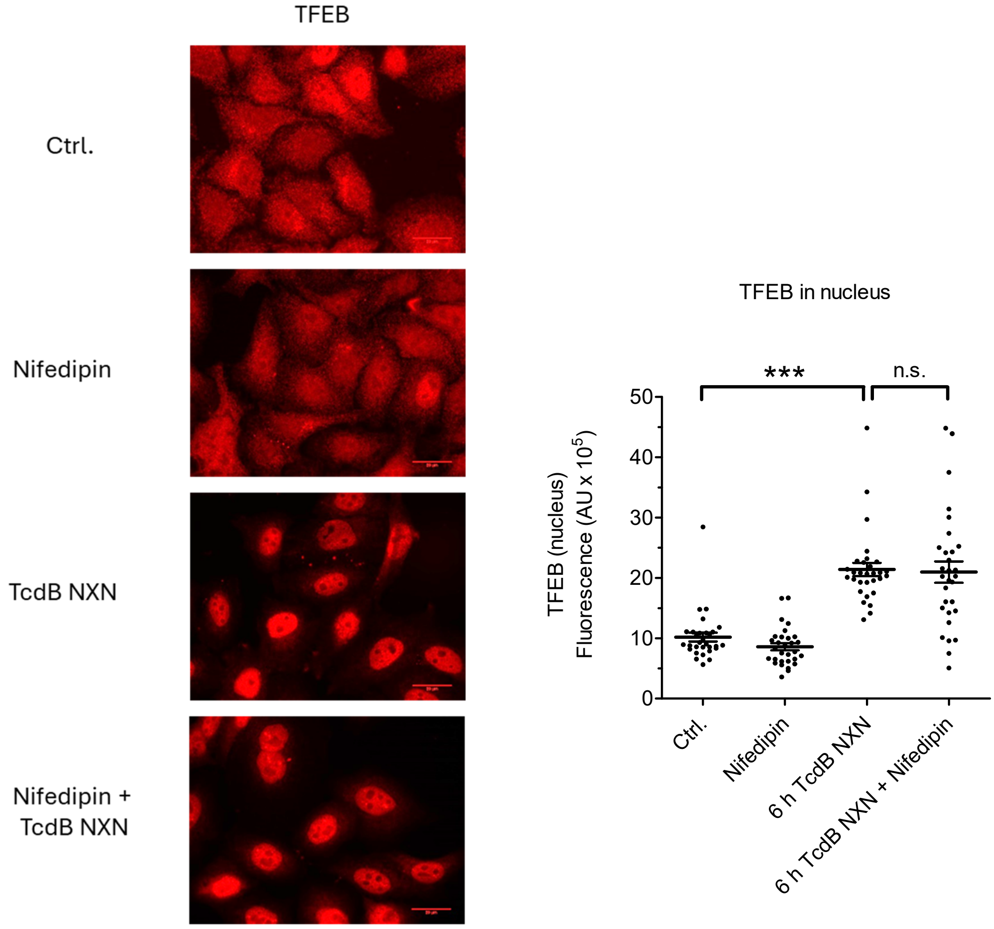

Left panel: Immunofluorescence staining of transcription factor EB (TFEB) in Hep-2 cells. TcdB NXN (1000 ng/µl) induced translocation of TFEB (AF594; red) from the cytosol to the nucleus after 6 h treatment. Preincubation of cells with 10 µM nifedipine for 30 min did not prevent toxin-induced translocation of TFEB to the nucleus. Scale bars represent 20 µm.

Right panel: Fluorescence in nucleus was quantified by ImageJ from each 10 cells of 3 replicates. Shown are mean values ± SEM, n = 30. *** = p-value <0.001, n.s. = non-significant, p-value >0.1

Fig. S6: Validation of GST-RILP pull-down assay for analyses of Rab7 activation at lower toxin concentration

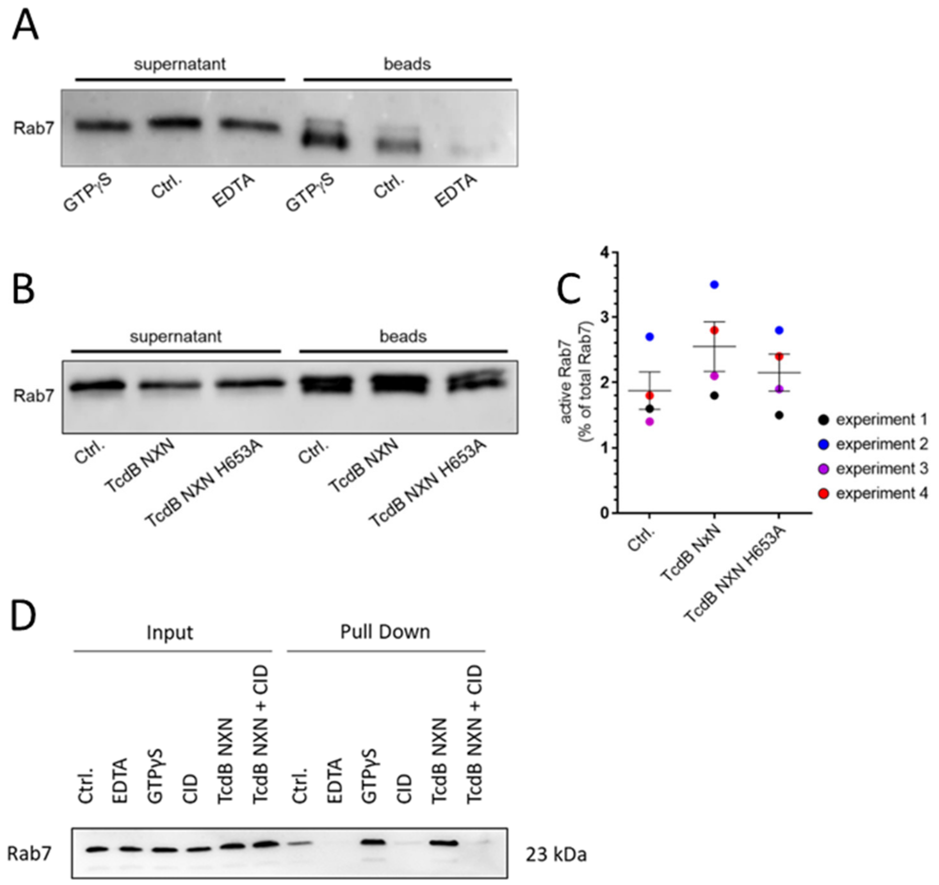

$Rab7_{GTP}$ pull down assays with GST-RILP.

Active Rab7 was measured by pull down assays with GST-RILP as described in methods. A) Pull down of $Rab7_{GTP}$ from cell lysate of untreated Hep-2 cells to validate method regarding activation and inactivation. Rab7 was activated by addition of non-hydrolyzable GTPγS or inactivated by EDTA to remove nucleotide. B) Pull down of active Rab7 from Hep-2 cell lysate of cells treated with 100 ng/ml TcdB NXN to avoid cell loss due to toxin-induced necrosis. TcdB NXN but not TcdB NXNN H653A showed more Rab7 in pull down assay. C) Densitometrical analyses of 4 separate pull down experiments. D) Representative pull down experiment shows inhibition of Rab7 by CID1067700 (CID). CID also prevented TcdB-induced increase in active Rab7 (TcdB NXN + CID) as shown in pull down. CID however did not prevent TcdB NXN-induced increase in total Rab7 as can be seen in input.
